# The Oxidative Stress Response in Elite Water Polo Players: Effects of Genetic Background

**DOI:** 10.1155/2017/7019694

**Published:** 2017-07-04

**Authors:** Mercurio Vecchio, Monica Currò, Fabio Trimarchi, Sergio Naccari, Daniela Caccamo, Riccardo Ientile, Davide Barreca, Debora Di Mauro

**Affiliations:** ^1^Department of Biomedical Sciences, Dental Sciences, and Morpho-Functional Imaging, Polyclinic Hospital University, Messina, Italy; ^2^Sport Center CUS UniME, University of Messina, Messina, Italy; ^3^Department of Chemical, Biological, Pharmaceutical and Environmental Sciences, University of Messina, Messina, Italy

## Abstract

Acute exercise is known to induce oxidative stress. Here we assessed the effects of gene polymorphisms SOD2 A16V, CAT −844 G>A, and GPx-1 rs1800668 C>T on oxidative stress markers in 28 elite water polo male players prior to and after a routinely programmed friendly match. The mean plasma concentrations of derivatives of reactive oxygen metabolites (dROMs), as well as lactic dehydrogenase (LDH) activity, creatine kinase (CK) activity, CK-MB, and myoglobin, were significantly increased after exercise, while blood antioxidant potential (BAP) and total free thiols were significantly decreased, compared with those measured before exercise. Advanced oxidation protein products (AOPP) were also increased after exercise but not significantly. We observed that water polo players having either AV16 or VV16 SOD genotype exhibited a significant increase of postexercise AOPP, LDH, CK, and myoglobin plasma levels in comparison with wild-type athletes. Water polo players having either CAT −844 GA or GPx1 CT genotype showed a significant increase of postexercise dROMs plasma levels and, respectively, GPx and CAT enzyme activities in comparison with wild-type subjects. These preliminary results suggest that the screening for gene variants of antioxidant enzymes could be useful to assess individual susceptibility to oxidative stress and muscle damage in water polo players.

## 1. Introduction

Exercise always stimulates a temporary change in redox balance towards a more oxidized state since active skeletal muscle cells continuously produce reactive oxygen and nitrogen species (ROS, RNS), as part of metabolic processes involving increased oxygen consumption [1]. Free radicals produced during exercise provoke a “hormetic” adaptive response to physical activity that is highly muscle fiber-specific and also positively modulate several physiological functions, such as cell signaling, immune response, and apoptosis [2, 3]. However, duration, intensity, and mode of exercise appear to affect differently the oxidative stress balance, and this may be dependent on age, gender, individual fitness levels, and nutritional status [4]. In particular, while regular exercise induces the upregulation of antioxidant as well as oxidative damage repairing systems [2, 5], acute exercise (“vigorous physical activity”) triggers a massive generation of ROS and RNS, also by anaerobic metabolism, with depletion in antioxidant defenses, and produces oxidative damage to proteins and DNA and secondary inflammation due to the phagocytic activity of immune cells [3, 6, 7]. These events result in varying degrees of mechanical and metabolic stress on the human body, finally leading to an impairment in cell and tissue functions [8]. Indeed, it is well known that oxidative stress is involved in the pathogenesis of hypertension, atherosclerosis, diabetes, and cancer and also accelerates aging process [9, 10].

The ROS-induced adaptive response following regular long-term training leads the upregulation of the antioxidant enzymatic systems, including superoxide dismutase (SOD), catalase (CAT), and glutathione peroxidase (GPX) that act synergistically with nonenzymatic antioxidants, that is, vitamins C and E, reduced glutathione, thiols, lipoic acid, and metallothioneins, to buffer the negative effects of oxidative stress [2, 11]. However, evidence has been provided by several investigations that exercise may positively or negatively affect oxidative status on the basis of training load, training specificity, and the basal level of training. In particular, the degree of oxidative damage and the time course for elevation in oxidative stress markers, during and following both acute aerobic and anaerobic exercise, resulted to be dependent on the type, intensity, volume, and duration of muscle contraction, leading to differences in the oxidative status between athletes in different sport disciplines [6, 8, 11–14].

For a long time, endurance training was considered the main cause of oxidative stress, but it was clarified that free radicals can also be produced through other pathways, which are not necessarily related to the oxygen demand. Indeed, several studies have shown that even the anaerobic exercise (high intensity training, weight lifting, etc.) can produce similar levels of oxidative stress [6]. Most of the studies regarding exercise-induced oxidative stress were carried out with exercise protocols including typical aerobic (running and cycling) and anaerobic (resistance training and sprints) exercise [15]. Intermittent exercises such as sporting games (soccer, handball, water polo, basketball, etc.) involve both aerobic and anaerobic metabolism and have been received small attention in the literature. Water polo is an example of intermittent (“interval”) sport, requiring a high fraction of oxygen consumption, and is composed by high intensity bursts of sprinting, each lasting between 7 to 14 seconds, interspersed with short periods of low to moderate intensity swimming [5].

Given the limited literature data available on oxidative stress response in water polo, we first investigated changes in oxidative stress biomarkers and antioxidant potential after a regular training program in a group of water polo male players. Moreover, we aimed to assess the effects of genetic background on oxidative response, with particular regard to the role played by single nucleotide polymorphisms (SNP) A16V of SOD2, encoding for intracellular Mn-SOD, −844 G>A of CAT, and rs1800668 of GPx-1.

## 2. Materials and Methods

### 2.1. Subjects

Twenty-eight Sicilian water polo male players participated in this study (age 25.1 ± 6.3 years; height 1.81 ± 0.07 m; body mass 85.5 ± 14.5 Kg; BMI 26.4 ± 4.4 Kg/m^2^; BSA 2.05 ± 0.16 m^2^). The players had at least 3 years of training and competition experience and took part in several competitions/year.

All participants completed medical history and exercise as well as lifestyle questionnaires to assess their current levels of physical activity and nutritional status. The mean consumption of fruits and vegetables in the whole study cohort was around 1 portion/day.

### 2.2. Training Routine

The recruited players followed a training routine of five days per week, with sessions lasting 4 h per day (2 h of swimming training and 2 h of water polo training, except Wednesday-match simulation). Indeed, the athletes trained five days per week, following a mesocycle preparation program. A mesocycle represents a phase of training with a duration of 2–6 weeks or microcycles, where the training program emphasizes the same type of physical adaptations, for example, muscle mass and anaerobic capacity. During the preparatory phase, a mesocycle commonly consists of 4–6 microcycles. The goal of the plan is to fit the mesocycles into the overall plan timeline-wise to make each mesocycle end on one of the phases and then to determine the workload and type of work of each cycle based on where in the overall plan the given mesocycle falls. The goal in mind is to make sure the body peaks for the high priority competitions by improving each cycle along the way.

### 2.3. Blood Collection

Blood sampling was performed at the end of the preseason training, after a preparatory phase of 12 weeks characterized by a high-volume, low-intensity training.

A blood sample of 4 mL was drawn from all participants 30 minutes prior to the match simulation, in a sitting position, from a forearm vein. A second blood sample was drawn 15 minutes after the simulated match (90 minutes of physical activity) from a forearm vein of the other arm. Several aliquots of whole blood, as well as plasma and serum fractions, obtained after centrifugation, were prepared and stored at −80°C until analysis.

The athletes or their parents, when needed, were informed about the aims of the study, and both provided a written informed consent form authorizing the present investigations. All procedures were conducted in accordance with the principles outlined in the Declaration of Helsinki for all human investigations.

### 2.4. Measurements of Derivatives of Reactive Oxygen Metabolites and Blood Antioxidant Potential

Serum levels of blood antioxidant potential (BAP) as well as derivatives of reactive oxygen metabolites (dROMs) were assessed by the use of commercially colorimetric assay kits (DIACRON Labs S.r.l.) according to manufacturer's instructions.

Reference values for dROMs (U CARR) were 300–320 borderline range; 321–340 low level of oxidative stress; 341–400 middle level of oxidative stress; 401–500 high level of oxidative stress; and >500 very high level of oxidative stress.

Reference values for BAP (*μ*mol/L) were >2200 optimum value; 2200–2000 borderline; 2000–1800 slight deficiency status; 1800–1600 deficiency status; 1600–1400 high deficiency status; <1400 very high deficiency status.

### 2.5. Measurements of Plasma Advanced Oxidation Protein Products

The concentrations of advanced oxidation protein products (AOPP) were analyzed by a colorimetric assay, using Chloramine T as standard, as described by Alagozlu and coworkers [16]. Reference limits for this method were <100 *μ*mol/L according to values reported in the general Caucasian population [16].

### 2.6. Assay of Free Thiols

The total amount of free thiols has been analyzed in plasma by colorimetric reactions under the conditions described by Turell and coworkers [17]. Reference range for this analysis was 400–600 *μ*mol/L.

### 2.7. Measurements of Antioxidant Enzyme Activities

The enzyme activities of superoxide dismutase (SOD), catalase (CAT), and glutathione peroxidase (GPx) were assessed before and after exercise.

Superoxide dismutase (SOD) activity was assayed according to the method of Paoletti and Mocali [18]. This assay is based on the oxidation of nicotinamide adenine dinucleotide reduced disodium salt mediated by a purely chemical reaction sequence which involves EDTA, Mn(II), mercaptoethanol, and molecular oxygen. The decrease of the rate of NADH oxidation is a function of enzyme concentration.

The measurement of CAT activity was carried out by measuring the rate of H_2_O_2_ breakdown at 240 nm according to the method of Luck [19]. Catalase activity was calculated based on the extinction coefficient of 43.1 M^−1 ^cm^−1^ for H_2_O_2_ at 240 nm and expressed as nmoles of H_2_O_2_ consumed/min/mg of protein.

GPx activity was assessed by using the commercial kit Glutathione Peroxidase Cellular Activity Assay available from Sigma Aldrich (Milan, Italy), according to manufacturer's instructions.

### 2.8. Measurements of Muscle Damage Markers

Plasma levels of lactic dehydrogenase (LDH) activity, creatine kinase (CK) activity, creatine kinase-MB (CK-MB), myoglobin (MGB), and troponin, as markers of muscle damage, were assessed by Siemens autoanalyzers (ADVIA 1800 and Centaur XP Siemens, Healthcare Diagnostics, Germany) using the appropriate reagent kit of the same company (Siemens, Healthcare Diagnostics, Germany). Reference ranges for these markers were LDH 140–280 U/L; CK ≤ 170 U/L; CK-MB ≤ 6.5 ng/mL; myoglobin ≤ 90 ng/mL; troponin ≤ 40 ng/mL.

### 2.9. Genotyping of SOD2, CAT, and GPX1 SNPs

Genomic DNA was extracted from peripheral blood lymphocytes samples using Gentra* Puregene* Blood Kit (Qiagen, Italy) according to manufacturer's instructions.

Genotyping for the SNPs SOD2 A16V (rs4880), CAT −844 G>A (rs769214), and GPx1 rs1800668 C>T was carried out by real-time PCR allelic discrimination in a 7500 Fast Real-Time PCR instrument (Applied Biosystems, Foster City, California, USA), using Predesigned TaqMan SNP Genotyping Assays (Applied Biosystems; assay ID: C_1202883_20, C_850486_20, C_2548962_20). Reaction conditions and thermal profile were the same reported by Gugliandolo and coworkers [20].

### 2.10. Statistical Analysis

Continuous data are expressed as mean ± standard deviation (SD) and the categorical variables as number and percentage. A paired sample* t*-test was used in order to compare the mean value of continuous variables reported before and after exercise.

Pearson's correlation was applied to assess the existence of any significant interdependence between numerical parameters. Compliance of genotype distribution to the Hardy-Weinberg equilibrium was estimated by Fisher's exact test, based on a Web program (http://ihg.gsf.de/cgi-bin/hw/hwa1.pl).

Univariate and multiple linear regression models were applied to verify the possible dependence of oxidative stress markers on genetic background.

To examine the effects of analyzed polymorphisms on continuous variables the one-way ANOVA followed by the Bonferroni post hoc analysis was performed. A *p* value ≤ 0.05 was considered statistically significant for all the analyses. Statistical analyses were performed using Statistic program v.7 for Window package.

## 3. Results

### 3.1. Biochemical Features of the Study Cohort

Plasma levels of redox markers, namely, dROMs, BAP, thiols, and AOPP, as well as enzyme activities of SOD, CAT, and GPX, were assessed in water polo players before and after exercise and are shown in Table 1. We observed that, in resting conditions, the mean levels of oxidated molecules (dROMs, AOPP) were increased, while antioxidant defenses (BAP, thiols) were reduced in comparison with normal reference ranges. This indicated a very high level of oxidative stress in water polo players recruited for this study. Postexercise mean plasma levels of dROMs, and those of BAP and total free thiols, were significantly higher and lower, respectively, than those before exercise. No significant changes after training were observed in mean plasma levels of AOPP as well as SOD, CAT, and GPx enzyme activities.

We also assessed the variations between preexercise and postexercise values in plasma levels of muscle damage markers, such as LDH, CK, CK-MB, myoglobin, and troponin (Table 1). Notably, at rest mean plasma concentrations of LDH were higher than normal reference values, while CK, CK-MB, myoglobin, and troponin levels were found to fall in the normal reference range. However, the levels of all muscle damage markers, except troponin, were found to be significantly increased after exercise in comparison with those measured before exercise.

The ratios between post- and preexercise values of redox markers were also calculated, and a correlation analysis was carried out to assess the relationships among the different markers. A significant positive correlation was found between the ratios of dROMs and those of AOPP (*R* = 0.44, *p* = 0.019), as well as dROMs and the muscle damage markers troponin (*R* = 0.587, *p* = 0.001) and myoglobin (*R* = 0.771, *p* = 0.000), and also between AOPP and LDH (*R* = 0.459, *p* = 0.014), SOD activity and LDH (*R* = 0.422, *p* = 0.025), CAT activity and CK (*R* = 0.473, *p* = 0.011), and LDH and CK (*R* = 0.409, *p* = 0.031). Instead, a significant negative correlation was observed between the ratios of dROMs and BAP (*R* = −0.48, *p* = 0.008), dROMs and thiols (*R* = −0.483, *p* = 0.009), and BAP and myoglobin (*R* = −0.644, *p* = 0.007).

### 3.2. Genetic Background of the Study Cohort

Genotype distributions of the SNPs SOD2 A16V, CAT −844 G>A, and GPx1 rs1800668 in water polo players recruited for this study were found to be in agreement with Hardy-Weinberg equilibrium (SOD2 *p* = 0.705; CAT *p* = 0.296; GPx1 *p* = 1.000).

Genotyping of recruited subjects for the SOD2 A16V polymorphism showed that the two alleles had the same frequencies in water polo players (A 0.50 versus V 0.50). The heterozygous AV16 genotype was the most represented among athletes, being found in 57.2% (*n* = 16) of the study cohort, while AA wild-type (21.4%; *n* = 6) and VV homozygous mutated subjects (21.4%; *n* = 6) had the same distribution.

A largely higher frequency was found for the CAT −844 G wild-type allele in comparison with the A mutated allele (0.79 versus 0.21). Notably, the −844 A mutated allele was only found in heterozygous state, with the GA heterozygous genotype being represented in 42.8% (*n* = 12) of the recruited subjects. The GG wild-type genotype was present in 57.2% (*n* = 16) of the study cohort.

The analysis of GPx1 rs1800668 genotype distribution pointed out that the C wild-type allele had a higher prevalence than the T mutated allele among the recruited water polo players (0.54 versus 0.46). The CT heterozygous genotype was the most frequent, being present in 50% (*n* = 14) of the studied population. As a whole, people bearing the T mutated allele accounted for over 70% of recruited water polo players, being also present in homozygous state in 21.4% (*n* = 6) of the study cohort. Wild-type subjects represented 28.6% (*n* = 8) of the study subjects.

### 3.3. Association between Genetic Background and Variability of Redox Markers

In order to understand the effects of genetic background on the variability of redox markers levels water polo players were grouped according to their genotype, and a between-groups comparison was carried out to assess differences in the plasma concentrations of redox markers, in resting conditions and after training (Table 2).

No significant differences were found among water polo players having different SOD2, CAT, and GPx1 genotype with regard to preexercise plasma levels of dROMs, BAP, and total thiols (Table 2). Moreover, AOPP plasma concentrations were not significantly different in individuals with different CAT and GPx1 genotype, while they were found to be significantly higher in water polo players having the SOD2 AV16 genotype than in those with VV genotype (Table 2). Notably, after 90 minutes of exercise AOPP concentrations were significantly increased not only in individuals bearing the SOD2 AV16 but also in those with VV16 genotype in comparison with those bearing the AA genotype (Table 2). After exercise, plasma values of dROMs were increased, while those of BAP and free thiols were concomitantly decreased, in water polo players having either heterozygous or homozygous mutated genotype for SOD2 V16A, CAT −844 G>A, and GPx1 rs1800668 polymorphisms compared with those having wild-type genotypes (Table 2). However, these differences were found to be significant only for subjects having either the CAT GA genotype or GPx1 CT genotype (Table 2), likely due to the small group size.

We also examined the influence of gene polymorphisms on the extent of redox marker variations following exercise that were calculated as the ratios between post- and preexercise values. Significant differences were observed only when analyzing the impact of the GPx1 rs1800668 on AOPP and dROMs variations. In particular, growing AOPP concentration ratios were observed in GPx1 TT mutated homozygous water polo players compared with athletes having other genotypes, while a significant increase in dROM ratios was found in TT or CT subjects compared with wild-type athletes.

Then, we analyzed the influence of different SOD2, CAT, and GPx1 genotypes on antioxidant enzyme activities (Table 3). In resting conditions significant differences were found only for water polo players having either the CAT −844 GA or GPx1 rs1800668 CT or TT genotype that exhibited greater SOD and GPx enzyme activities in comparison with wild-type athletes. Instead, all the examined polymorphisms did not affect CAT enzyme activity. Interestingly, the CAT-844 G>A genotype was also shown to be associated with a significant increase of postexercise GPX enzyme activity in comparison with wild-type CAT genotype GG, and the GPx1 rs1800668 CT genotype resulted to be associated with significantly increased postexercise CAT enzyme activity in comparison with wild-type GPx1 CC genotype. Surprisingly, the GPx1 rs1800668 TT genotype was associated with a significant reduction in postexercise GPx and CAT enzyme activities in comparison with wild-type GPx1 CC genotype. However, these results should be considered with caution given the small number of homozygous subjects that may be not representative of actual variations (Table 3).

Finally, we analyzed the influence of different SOD2, CAT, and GPx1 genotypes on the variability of muscle damage markers (Table 4). In resting conditions significant differences were found only for water polo players having either the SOD AV16 genotype or the VV16 genotype that exhibited greater myoglobin or greater troponin, myoglobin, and CK-MB values, respectively, in comparison with wild-type athletes. Instead, the CAT −844 G>A and the GPx1 rs1800668 C>T did not affect plasma concentrations of muscle damage markers. After exercise we found that athletes with SOD2 AV16 genotype had higher LDH concentrations than those with AA or VV genotype, while athletes having the SOD VV16 genotype had significantly higher concentrations of CK and myoglobin in comparison with wild-type AA or heterozygous AV ones (Table 4).

## 4. Discussion

Several investigations provided evidence that exercise may positively or negatively affect oxidative status on the basis of training load, training specificity, and the basal level of training. In particular, the degree of oxidative damage and the time course for elevation in oxidative stress markers, during and following both acute aerobic and anaerobic exercise, result to be dependent on the type, intensity, volume, and duration of muscle contraction, and also on gender, age, individual fitness levels, and nutritional status, leading to differences in the oxidative status between athletes in different sport disciplines [4, 8, 12, 13].

A water polo match is characterized by high intensity intermittent activity with periods of intensive muscular activity followed by periods of moderate exercise or even rest, which involves both aerobic and anaerobic metabolism [5]. In elite female water polo players it has been observed that the concentration of blood biomarkers of oxidative stress and inflammation varies over the course of an agonistic season [21]. Our results demonstrated that, at rest, athletes exhibited dROM, BAP, and AOPP plasma levels, representing valuable markers of oxidative stress, outside of the desirable concentration ranges, indicating an imbalance between prooxidants and antioxidants. Interestingly, AOPP plasma concentrations were higher than those previously observed in a group of elite hurdlers [22], in agreement with the suggested influence of training level and mode on oxidative stress. The high level of oxidative stress found in resting athletes, recruited for this study, was likely a consequence of the high-volume training required during the mesocycle preparatory phase. In addition, after training we observed a further increase in dROM values and a reduction in total antioxidant capacity in comparison to rest values. Training session of team sports and/or match may also lead to an inflammatory response associated with muscle injury due to eccentric contraction used in these sports [23]. Notably, intensified training was shown to induce a biphasic response of total antioxidant capacity, represented by a significant increase after low- and high-volume training, and a decline after very high-volume training [3]. This condition, together with weekly competition and a diet at low intake of fruits and vegetables, impeded the normal recovery of athletes and may play a major role in the development of chronic oxidative stress and cellular damage [13].

It has been hypothesized that SOD, CAT, and GPx, which represent the major enzymatic antioxidant systems involved in ROS scavenging, display different features of adaptation to training. Both CAT and GPx convert hydrogen peroxide (H_2_O_2_) produced by SOD to water and oxygen, but the threshold of H_2_O_2_ for their induction is different. During moderate exercise GPx activity is enough to scavenge low levels of produced H_2_O_2_, while after strenuous exercise there is an excessive production of H_2_O_2_ leading to an increase in CAT synthesis to compensate for the insufficient clearance of H_2_O_2_ by GPx [24].

In our study, although we observed a reduction in BAP levels after training, the evaluation of SOD, CAT, and GPx activity did not reveal significant differences between pre- and postexercise values. This could be explained by the fact that the enzymatic antioxidants are mainly present intracellularly, while nonenzymatic antioxidants prevail at extracellular level. Indeed, it has been shown that, in condition of strenuous and continuous training, variations of antioxidant parameters are different. As example, erythrocyte SOD, GSH-Px activities, and blood GSH were shown to be unchanged, while plasma total antioxidant status was decreased, and plasma GSH-Px activity increase failed to prevent oxidative exercise-induced damage in overloaded triathletes [13]. This suggests nonsynergistic responses in antioxidant parameters during OT (even if some parameters eventually adapt, it may be insufficient to prevent oxidative damage).

We also analyzed levels of LDH, CK, and CK-MB that have been confirmed as useful serum markers of muscle injury. During exercise the increase in ROS production can induce lipid peroxidation which in turn alters membrane permeability causing the release of protein from myocells. Previous animal studies reported a positive correlation between increased lipid peroxidation and CK as well as aspartate-aminotransferase, suggesting that biomarkers of muscle damage during exercise are indicative of oxidative stress [4]. Serum CK concentrations may change in response to eccentric exercise too. Water polo is considered a noneccentric exercise mode; nevertheless there is evidence of relevant cocontraction muscular intervention modes, possibly implying also eccentric actions of the antagonist muscle [25]. An adaptive behavior of trained muscle depending on type and duration of exercise has been demonstrated. Peak values in serum CK have been recorded after endurance event [26] as well as in eccentric exercise [27]. Also markers of myocardial damage, such as the CK-MB isoenzyme, myoglobin, and cardiac troponins, are affected by prolonged or strenuous exercise [28]. However, Dahlqvist et al. [29] suggested that cardiac troponin represents a marker of more severe alterations associated with sarcomeric damage. Indeed, it has been demonstrated that cardiac troponins appear in blood of well-trained athletes competing in ultra-endurance events such as Ironman Triathlon or exercise of even longer duration, while they are not detected in blood after intermittent or short duration (<90 min) exercise [30, 31]. Accordingly, in water polo players after training we reported an increase in LDH, CK, CK-MB, and myoglobin plasma levels, while no changes were observed for troponin. These results indicate that intermittent high intensity activity in water polo athletes causes a moderate muscle injury.

The muscle injury is dependent on oxidative stress as shown by the positive correlation found between increased dROMs as well as AOPP and increased troponin, myoglobin, and LDH, and also between increased SOD activity as well as CAT activity and increased LDH and CK. Instead, high levels of BAP result to be protective against muscle injury, as shown by the negative correlation found between BAP and myoglobin.

In this study, we also examined the effects of gene polymorphisms in antioxidant enzymes, namely, SOD2 A16V, CAT G-844A, and GPx1 rs1800668, on oxidative stress response in water polo players.

It has been shown that the A16 variant allows a 30–40% more efficient targeting of MnSOD to the mitochondria than V16 and therefore is associated with high levels of mitochondrial concentration and activity [32]. In this study we observed higher levels of oxidative stress before and after exercise in athletes bearing the mutated allele V16, either in heterozygosis or in homozygosis, than in wild-type subjects, as shown by increased dROMs and AOPP and decreased BAP and free thiols. However, the differences with wild-type subjects were found to be significant only for variations of AOPP plasma levels. Differences in SOD2 A16V genetic background did neither affect plasma SOD activity nor affect CAT or GPx activities. This could be explained taking into account the fact that SOD2 represents the primary level of antioxidant defense in cellular mitochondria. Instead, the SOD2 A16V polymorphism represents a risk factor for increased muscle injury in water polo players, since athletes bearing the homozygous mutated VV16 genotype exhibited higher values of LDH, CK, CK-MB, troponin, and myoglobin than wild-type AA and heterozygous AV subjects, even if these differences were not always significant likely due to the small size of genotype subgroups.

The CAT G-844A polymorphism may influence CAT transcription by modulation of the transcriptional factor binding position, and it has been postulated that the A allele is associated with decreased CAT activity in comparison with the G allele [33]. Thus, it is reasonable to hypothesize that individuals bearing this CAT polymorphism are more susceptible to oxidative stress as they possess lower levels of CAT protein than wild-type subjects. Given the lack of AA homozygous subjects in the investigated population we could not fully evaluate the effect of this polymorphism on oxidative stress response. However, our results show that heterozygous GA water polo players had higher levels of oxidative stress than wild-type subjects, even if significant differences were only found for dROMs levels after exercise, probably due to the small size of sampled subgroups. These results were also confirmed by the examination of antioxidant enzyme activities, since heterozygous subjects had a significantly higher GPx activity before exercise, and significantly higher GPx and SOD activities after exercise. However, differences in CAT G-844A genetic background among the recruited water polo players did not affect the variability of muscle damage markers before and after exercise.

GPx activity represents the second line of enzymatic antioxidant defense, and Gpx1, one of six isoforms of GPx, is widely present in human cells and in vascular endothelium too [34]. The GPx1 rs1800668 (C>T) polymorphism has been reported to affect GPx1 gene transcription rate and could reasonably play a role in increased individual susceptibility to oxidative stress. Water polo players having the T mutated allele had pre- and postexercise increased levels of prooxidant markers (dROMs, AOPP) and decreased levels of antioxidant markers (BAP, thiols), even if significant differences were only found for dROMS after exercise, likely due to the small size of genotype subgroups. Our preliminary results suggest that the GPx1 rs1800668 polymorphism plays a major role in modulating antioxidant enzyme activities, since both SOD and CAT activities were significantly increased in heterozygous CT water polo players compared with wild-type subjects. Instead, this GPx1 polymorphism seems to play a minor role in muscle damage given that although water polo players having the CT heterozygous genotype showed the highest levels of LDH, CK, and myoglobin after exercise, these differences were not statistically significant in comparison with wild-type subjects.

## 5. Conclusions

Due to relative small number of water polo players recruited, the results of our study should be considered preliminary; then further investigations are required, for example, in order to examine the combined effects of all three polymorphisms in different haplotypes.

Moreover, the data on pre- and postexercise variations of redox biochemical parameters need to be more carefully evaluated. In fact, a correct standardization on timing and way of blood samples collection should be developed. For example, assessing redox status at beginning and end of season could be useful to monitor the real variations in oxidative stress parameters and the possible overtraining status in water polo players.

Information obtained may be used by coaches and/or athletic trainers to enhance recovery, prevent training overload, and optimize athletic performance.

## Figures and Tables

**Table 1:** Biochemical features of water polo players recruited for this study.

	Preexercise	Postexercise	*p value*	*Reference values*
dROMs (U Carr)	656.1 ± 97.35	745.8 ± 138.2	0.001^*∗∗*^	<320
AOPP (*µ*g/L)	251.3 ± 39.04	236.3 ± 45.9	0.133	<100
BAP (*µ*mol/L)	1545.9 ± 531.7	655.7 ± 223.8	<0.001^*∗∗∗*^	>2200
Total free thiols (*µ*M)	344.3 ± 44.4	327.5 ± 39.5	0.046^*∗*^	400–600
SOD activity (U/mg)	1.537 ± 0.313	1.425 ± 0.365	0.174	—
CAT activity (H_2_O_2_ nmoles consumed/min/mg of protein)	0.572 ± 0.265	0.578 ± 0.216	0.917	—
GPx activity (U/mg)	0.037 ± 0.006	0.034 ± 0.009	0.103	—
LDH (U/L)	347.1 ± 37.4	369.5 ± 49.0	0.001	140–280
CK (U/L)	160.5 ± 58.8	219.3 ± 74.0	0.000	≤170
CK-MB (ng/mL)	1.2 ± 0.3	1.3 ± 0.2	0.004	≤6.5
Troponin (pg/mL)	6.8 ± 1.1	6.8 ± 0.9	1.000	≤40
Myoglobin (ng/mL)	39.2 ± 11.8	92.3 ± 24.3	0.000	≤90

Biochemical data are reported as mean ± SD. ^*∗*^*p* < 0.05 significant value in comparison with preexercise values; ^*∗∗*^*p* < 0.01, significant value in comparison with preexercise values; ^*∗∗∗*^significant value in comparison with preexercise values. Statistical differences were evaluated using a* paired sample t-test.*

**Table 2:** Variability of oxidative stress markers in water polo players having different SOD2, CAT, and GPx1 genotypes.

Genotype	dROMs (UCarr)		BAP (*µ*mol/L)		Thiols (*µ*M)		AOPP (*µ*g/L)	
	*Preexercise*	*Postexercise*	*Preexercise*	*Postexercise*	*Preexercise*	*Postexercise*	*Preexercise*	*Postexercise*
*SOD2 A16V*								
AA (*n* = 6)	608.8 ± 139.7	728.9 ± 65.6	1756.0 ± 739.1	664.5 ± 92.9	0.35 ± 0.01	0.31 ± 0.04	204.3 ± 32.8	203.9 ± 16.4
AV (*n* = 16)	645.5 ± 70.5	737.9 ± 170.3	1362.7 ± 630.2	590.6 ± 238.8	0.33 ± 0.05	0.33 ± 0.03	263.6 ± 35.4^#^	232.5 ± 44.3^#^
VV (*n* = 6)	692.0 ± 72.0	750.9 ± 101.9	1894.1 ± 209.5	831.5 ± 207.8	0.37 ± 0.01	0.36 ± 0.03	257.3 ± 36.3	284.1 ± 25.6^##^
*CAT G-844A*								
GG (*n* = 16)	635.9 ± 104.6	694.4 ± 149.1	1472.0 ± 649.9	670.6 ± 277.6	0.33 ± 0.05	0.33 ± 0.04	255.3 ± 33.0	242.5 ± 53.3
GA (*n* = 12)	683.0 ± 83.5	814.3 ± 87.2^§^	1644.5 ± 315.1	635 ± 130.7	0.35 ± 0.03	0.32 ± 0.03	245.9 ± 46.9	228.1 ± 34.1
AA (*n* = 0)	/		/		/		/	
*GPx1 rs1800668*								
CC (*n* = 8)	617.3 ± 90.1	607.5 ± 152.2	1255.1 ± 752.5	668.0 ± 376.1	0.32 ± 0.05	0.35 ± 0.04	260.7 ± 25.9	233.3 ± 66.7
CT (*n* = 14)	693.5 ± 55.5	819.5 ± 85.9^*∗∗∗*^	1664.2 ± 418.6	645.9 ± 164.9	0.36 ± 0.04	0.31 ± 0.03	244.5 ± 42.5	238.1 ± 36.9
TT (*n* = 6)	620.6 ± 154.9	758.3 ± 74.1	1657.8 ± 298.0	662.1 ± 32.4	0.34 ± 0.02	0.34 ± 0.03	254.7 ± 48.1	236.1 ± 39.8

^#^ *p* = 0.019 and ^##^*p* = 0.011 statistical differences in comparison to SOD2 AA16 genotype; ^§^*p* = 0.02 significant difference in comparison to *CAT GG-844* genotype; ^*∗∗∗*^*p* = 0.000 significant difference in comparison to athletes carrying the *GPx1 CC* genotype.

**Table 3:** Variability of antioxidant enzyme activities in water polo players having different SOD2, CAT, and GPX1 genotypes.

Genotype	SOD activity (U/mg)		CAT activity (nmoles of H_2_O_2_ consumed/min/mg of protein)		GPX activity (U/mg)	
	*Preexercise*	*Postexercise*	*Preexercise*	*Postexercise*	*Preexercise*	*Postexercise*
*SOD2 A16V*						
AA (*n* = 6)	1.64 ± 0.07	1.38 ± 0.39	0.50 ± 0.07	0.64 ± 0.19	0.040 ± 0.000	0.040 ± 0.000
AV (*n* = 16)	1.58 ± 0.28	1.53 ± 0.39	0.55 ± 0.32	0.56 ± 0.24	0.037 ± 0.006	0.003 ± 0.010
VV (*n* = 6)	1.30 ± 0.43	1.19 ± 0.10	0.70 ± 0.18	0.56 ± 0.20	0.033 ± 0.005	0.033 ± 0.010
*CAT-844 G>A*						
GG (*n* = 16)	1.43 ± 0.39	1.49 ± 0.37	0.54 ± 0.19	0.56 ± 0.26	0.035 ± 0.007	0.031 ± 0.011
GA (*n* = 12)	1.67 ± 0.09^#^	1.33 ± 0.36	0.61 ± 0.34	0.60 ± 0.14	0.040 ± 0.000^#^	0.038 ± 0.004^#^
AA (*n* = 0)	/	/	/	/	/	/
*GPX1 rs1800668*						
CC (*n* = 8)	1.27 ± 0.48	1.42 ± 0.25	0.46 ± 0.17	0.49 ± 0.26	0.0325 ± 0.046	0.030 ± 0.013
CT (*n* = 14)	1.61 ± 0.10^§^	1.56 ± 0.41	0.55 ± 0.18	0.70 ± 0.15^§^	0.0400 ± 0.0055^§§^	0.034 ± 0.007
TT (*n* = 6)	1.70 ± 0.12^§^	1.11 ± 0.16^*∗*^	0.77 ± 0.42	0.41 ± 0.09^*∗∗*^	0.0367 ± 0.0051	0.040 ± 0.000

^#^ *p* < 0.05, significant difference in comparison to athletes carrying the CAT GG-844 genotype; ^§^*p* < 0.05, significant difference in comparison to athletes carrying the GPX1 CC genotype; ^§§^*p* = 0.010, significant difference in comparison to GPX1 CC genotype; ^*∗*^*p* < 0.05, significant difference in comparison to athletes carrying the GPX1 CT genotype; ^*∗∗*^*p* = 0.01, significant difference in comparison to athletes carrying the GPX1 CT genotype.

**Table 4:** Variability of muscle damage markers in water polo players having different SOD2, CAT, and GPX1 genotypes.

*Genotype distribution*	LDH (U/L)		CK (U/L)		CK-MB (ng/mL)		Troponin (pg/mL)		Myoglobin (ng/nL)	
	Preexercise	Postexercis	Preexercise	Postexercis	Preexercise	Postexercis	Preexercise	Postexercis	Preexercise	Postexercis
*SOD2 A16V*										
AA (*n* = 6)	356.5 ± 20.5	428.0 ± 20.9	174.7 ± 62.3	220.8 ± 47.9	1.37 ± 0.34	1.46 ± 0.29	7.3 ± 0.5	6.7 ± 1.0	45.5 ± 11.4	96.4 ± 11.6
AV (*n* = 16)	333.2 ± 39.9	338.2 ± 21.8^###^	138.8 ± 41.5	184.2 ± 39.7	1.05 ± 0.29	1.22 ± 0.25	6.4 ± 1.2	6.6 ± 0.9	32.1 ± 8.7^#^	76.6 ± 9.7
VV (*n* = 6)	375.0 ± 26.1	420.2 ± 46.7	204.3 ± 74.7	311.5 ± 91.4^#,*∗∗∗*^	1.42 ± 0.18^*∗∗*^	1.37 ± 0.08	7.7 ± 0.5^*∗*^	7.7 ± 0.5	47.0 ± 10.9^*∗*^	119.5 ± 30.5^*∗∗∗*^
*CAT-844 G>A*										
GG (*n* = 16)	346.1 ± 42.9	369.1 ± 50.6	161.6 ± 67.2	228.9 ± 90.1	1.28 ± 0.26	1.32 ± 0.21	7.0 ± 1.0	7.0 ± 0.9	42.1 ± 10.2	100.4 ± 28.9
GA (*n* = 12)	348.5 ± 30.2	370.0 ± 48.9	159.2 ± 48.4	206.6 ± 45.3	1.09 ± 0.38	1.28 ± 0.30	6.7 ± 1.3	6.7 ± 0.1	36.2 ± 13.2	84.2 ± 16.8
AA (*n* = 0)	/		/		/		/		/	/
*GPX1 rs1800668*										
CC (*n* = 8)	341.9 ± 49.4	367.4 ± 61.9	138.0 ± 49.0	212.0 ± 52.8	1.35 ± 0.30	1.35 ± 0.30	7.5 ± 0.5	7.2 ± 0.9	37.3 ± 7.8	81.3 ± 6.5
CT (*n* = 14)	346.9 ± 35.3	373.9 ± 53.2	180.8 ± 70.1	234.1 ± 95.2	1.08 ± 0.30	1.07 ± 0.30	6.6 ± 1.2	6.6 ± 0.9	41.4 ± 16.1	99.1 ± 33.4
TT (*n* = 6)	354.8 ± 27.8	362.0 ± 10.2	143.2 ± 15.4	194.7 ± 25.5	1.28 ± 0.35	1.28 ± 0.36	6.7 ± 1.4	7.0 ± 0.9	36.5 ± 1.6	89.8 ± 3.9

^#^ *p*<0.05, statistical difference in comparison to SOD2 AA16 genotype; ^###^*p*= 0.001, significant differences in comparison to SOD2 AA16 and VV16 genotypes; ^*∗*^*p*<0.05, ^*∗∗∗*^*p*<0.001, significant difference in comparison to SOD2 AV16 genotype.
